# Antibacterial Residue Excretion via Urine as an Indicator for Therapeutical Treatment Choice and Farm Waste Treatment

**DOI:** 10.3390/antibiotics10070762

**Published:** 2021-06-23

**Authors:** María Jesús Serrano, Diego García-Gonzalo, Eunate Abilleira, Janire Elorduy, Olga Mitjana, María Victoria Falceto, Alicia Laborda, Cristina Bonastre, Luis Mata, Santiago Condón, Rafael Pagán

**Affiliations:** 1 Instituto Agroalimentario de Aragón-IA2, Universidad de Zaragoza-CITA, 50013 Zaragoza, Spain; mjserran@unizar.es (M.J.S.); Diego.Garcia@unizar.es (D.G.-G.); omitjana@unizar.es (O.M.); vfalceto@unizar.es (M.V.F.); alaborda@unizar.es (A.L.); cbonastr@unizar.es (C.B.); scondon@unizar.es (S.C.); 2 Public Health Laboratory, Office of Public Health and Addictions, Ministry of Health of the Basque Government, 48160 Derio, Spain; e-abilleira@euskadi.eus (E.A.); j-elorduy@euskadi.eus (J.E.); 3 Department of R&D, ZEULAB S.L., 50197 Zaragoza, Spain; lmata@zeulab.com

**Keywords:** antibiotic, sulfonamide, quinolone, excretion, LC–MS/MS, urine

## Abstract

Many of the infectious diseases that affect livestock have bacteria as etiological agents. Thus, therapy is based on antimicrobials that leave the animal’s tissues mainly via urine, reaching the environment through slurry and waste water. Once there, antimicrobial residues may lead to antibacterial resistance as well as toxicity for plants, animals, or humans. Hence, the objective was to describe the rate of antimicrobial excretion in urine in order to select the most appropriate molecule while reducing harmful effects. Thus, 62 pigs were treated with sulfamethoxypyridazine, oxytetracycline, and enrofloxacin. Urine was collected through the withdrawal period and analysed via LC-MS/MS. Oxytetracycline had the slowest rate of degradation (a half-life time of 4.18 days) and the most extended elimination period in urine (over 2 months), followed by enrofloxacin (a half-life time of 1.48 days, total urine elimination in ca. 3 weeks) and sulfamethoxypyridazine (a half-life time of 0.49 days, total urine elimination in ca. 1 week). Bacterial sensitivity and recommendations for responsible use are limiting when selecting the treatment. Nevertheless, with similar effectiveness, sulfamethoxypyridazine would be the choice, as waste treatment would only need to be implemented for 1 week after treatment. Thus, more in-depth knowledge regarding antibacterial elimination would improve resource management, while protecting animals and consumers’ health.

## 1. Introduction

Antimicrobials are compounds administered to livestock for a number of reasons. On the one hand, they have traditionally been administered for non-therapeutic purposes, including disease prophylaxis and growth promotion [1,2]; on the other hand, they can be dispensed to treat diseases when they emerge. The diversity of uses for antimicrobials in farm breeding has led to an increased worldwide demand for antibiotics in livestock production which has even surpassed the total amount of human consumption [3]. As a consequence, it is now encouraged to rationalize their use [4,5]. Furthermore, the EMA (European Medicines Agency) has issued recommendations regarding their order of eligibility [6]. Indeed, growth promoters have been banned in the European Union since 2006, and prophylaxis is now likewise restricted in some animal groups (Regulation (EU) 2019/4 [7] and Regulation (EU) 2019/6 [8]). Nevertheless, the severity of some illnesses still requires treatment with antimicrobials.

Ideally, disease management would involve an accurate diagnosis of the causal agent involved, through the collection of an accurate sample for bacterial culture and antimicrobial susceptibility testing. Nonetheless, susceptibility is not the only feature to be considered, as certain antimicrobials are limited to human use [6], and the mode of administration of certain other antimicrobials might not be feasible on each type of farm. After administration, antimicrobials spread throughout the animal’s organism, reaching most of its tissues. Nevertheless, the degree of concentration in each organ depends on the antibiotic’s pharmacokinetics as well as the moment of treatment. Once the compound has fulfilled its function, it leaves the tissues and is excreted through several pathways, of which urine is one of the most predominant [9,10,11,12,13].

Although antimicrobial therapy significantly reduces economic loss and mortality rates on farms, antimicrobial residues produce a considerable array of negative side effects when they are released to the environment. On the one hand, antimicrobial residues exert a series of potential long-term adverse effects on humans and animals [14,15,16,17]. On the other, such deposits wield an intense pressure towards the genetic selection of resistant bacteria [18,19]. Although direct damage on human health is the most visible consequence, the development of resistant bacteria populations is the most severe side effect, as antimicrobials become useless against common illnesses easily addressed before. So severe is this problem that, by the year 2050, antimicrobial resistance generation (AMR) is estimated to be causing 10 million deceases per year overpassing illnesses such as cancer [4].

Several studies have set out to determine the amount of antimicrobial residues in manure and slurry and have found concentrations as high as 20,000 µg kg^−1^ of sulfonamide in Switzerland [20], 43 µg kg^−1^ of tiamulin in Germany [21], and 31 different antibiotic residues in China, with concentrations of sulfamethazine exceeding 5650 µg kg^−1^ [22]. Manure and slurry are usually used as natural fertilizers in agriculture, thereby dramatically spreading antibacterial residues in nature [23,24]. They are washed from the soil by rainwater, with the possibility of reaching nearby streams [25,26,27]. The United States Geological Survey (USGS) recovered 21 different antibiotics from watercourses in 1999—2000 [28].

Once these compounds have reached the environment, it is no longer feasible to eliminate them because some of them are not biodegradable [29]. The best way to manage them is by their destruction before they leave the farm. Several methods are being studied to avoid antibiotic release, such as heating [30,31], sorption of antibiotics onto carbonaceous materials [18], anaerobic digestion [32,33], manure composting [34,35], solid-liquid separation [36], and Fenton reaction [37]. These procedures involve a series of technologies, the choice of which will be dependent on the antimicrobial used and the concentration excreted, which, in turn, will depend on the moment of therapy. Moreover, an antimicrobial will be chosen for its effectiveness and for its compliance with European recommendations. Hence, this study’s objective is to describe the rate of antimicrobial excretion in urine after treatment with a series of different antimicrobials (a tetracycline, a sulfonamide, and a quinolone) in order to suggest which is the most appropriate molecule for therapeutical treatment, thereby guaranteeing a successful therapy along with the most thorough elimination of antimicrobial residues, to ensure sustainable resource management.

## 2. Results and Discussion

Antibacterial therapy is widespread and goes hand in hand with our current welfare state and economic development. Once antibiotics exert their effect, urine has been demonstrated to be the main path of elimination of most of their residues. Unexpected delivery of these compounds to external areas such as farm surroundings or the food chain [21] might exacerbate the serious problem of the emergence of AMR. One of the easiest ways of limiting the entrance of antibacterial compounds into the environment is by treating animal wastes before their removal from farm facilities [38]. Thus, better knowledge on the subject of antimicrobial elimination would rationalize production thanks to better resource management in waste treatment and by helping producers to choose the most suitable antibacterial treatment. A detailed study of the excretion rates of antimicrobials via urine would represent a first step in helping to make the best decisions when planning waste management. Thus, as a first step, 62 pigs were treated with several antimicrobials currently used in livestock farming (sulfamethoxypyridazine, oxytetracycline, and enrofloxacin). After collection, urine samples were analysed using LC-MS/MS within the first month, as certain antimicrobials might otherwise soon have degraded [39,40]. Novel extraction procedures and analysis techniques were developed in order to perform accurate analysis.

### 2.1. Excretion of Sulfamethoxypyridazine via Urine

Sulfamethoxypyridazine is a bacteriostatic compound from the sulfonamide family which, once administrated, spreads widely throughout the body. After attaining therapeutic concentrations in the bloodstream and having reached body tissues, it is mainly excreted through the kidneys, although it can be excreted to a lesser extent via fluids other than urine [41]. It can be classified as a long-acting sulfonamide, which is why the withdrawal period of sulfamethoxypyridazine-based drugs for food-producing animals is quite extended (Table 1).

**Figure 1:**
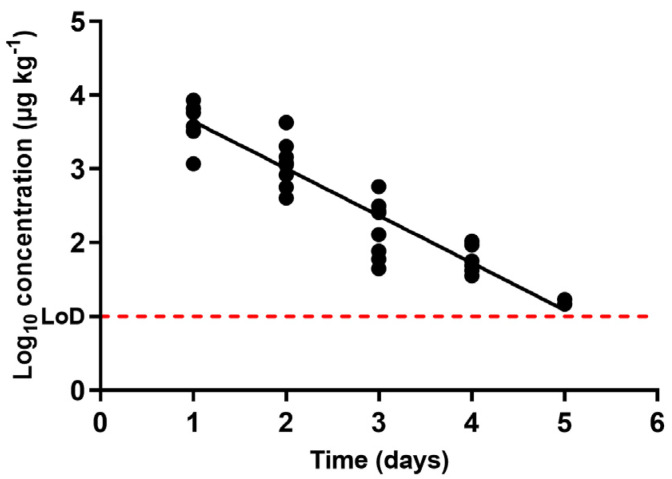
Occurrence of the concentration of sulfamethoxypyridazine in urine samples obtained from pigs treated with sulfamethoxypyridazine at pre-set intervals within the withdrawal period (when day 0 matches the end of the treatment), determined via liquid chromatography with tandem mass spectrometry (LC-MS/MS). The LoD dotted line represents the detection limit of the analytical technique for sulfamethoxypyridazine.

Figure 1 shows the occurrence of sulfamethoxypyridazine concentration in urine through the withdrawal period. As expected, this compound was present in higher concentrations within the first days, decreasing at an exponential rate as the withdrawal period progressed until reaching concentrations that approached the LoD of the chromatographic technique. The decrease of sulfamethoxypyridazine in urine to levels as low as the LoD (10 ppb) ensures almost complete excretion. From these data, and according to Equation (1), this is equivalent to a half-life time of 0.49 days (Table 1).

Figure 1 also displays somewhat wide variations among the elimination rates in individual animal specimens. Although this variability between animals subjected to the same treatment and withdrawal period could be viewed as large (as reflected in the *R^2^* values of the regression (Table 1)), differences between rates of removal are ascribable to individual variations. These pharmacokinetic differences are also consistent with the ones previously described for other antimicrobials such as macrolides [42] or even for sulfonamide pharmacokinetics after treatment via either oral [43] or intravenous administration [44].

According to the regression carried out, the time period required to complete the excretion of sulfamethoxypyridazine by urine could be estimated at almost a week (Table 1). Although sulfamethoxypyridazine is rapidly eliminated, this antimicrobial family is nevertheless commonly found in high levels in farm wastes [45], and thus, waste treatments are required for at least seven days after the end of treatment, and their features will be dependent on the concentration of the excreted sulfonamide.

### 2.2. Excretion of Oxytetracycline via Urine

Oxytetracycline is commonly administered via intramuscular injection and usually has long-lasting formulations: the drug is slowly released through the body over a prolonged period of time, and this behaviour is reflected in certain studies which detected long mean residence times [46], which correspond with the extended withdrawal period set by manufacturers for commercial oxytetracycline-based products (Table 1). Regarding its excretion pathways, 60% of the antibiotic administered in the treatment is excreted in urine via glomerular filtration, while the remaining 40% is eliminated in the faeces [9].

**Figure 2:**
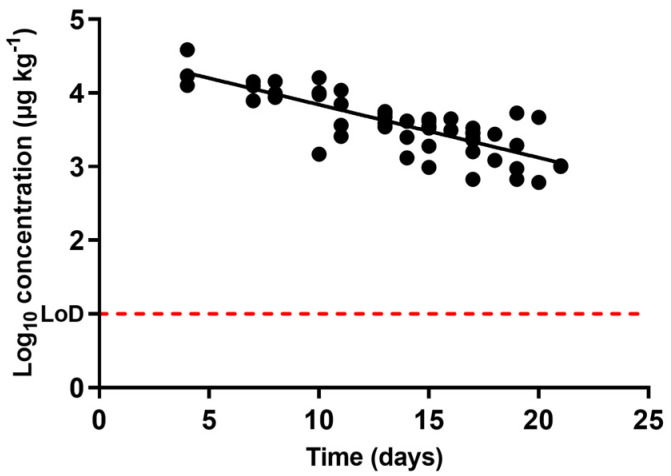
Occurrence of the concentration of oxytetracycline in urine samples obtained from pigs treated with oxytetracycline at pre-set intervals within the withdrawal period (when day 0 matches the end of the treatment), determined by liquid chromatography with tandem mass spectrometry (LC-MS/MS). The LoD dotted line represents the detection limit of the analytical technique for oxytetracycline.

Figure 2 shows the occurrence of oxytetracycline concentration in urine through the withdrawal period. It shows that the antimicrobial concentration in urine decreased as the withdrawal period progressed, and the compound was depleted following an exponential rate. Once more, certain notable differences between the rates of excretion of oxytetracycline via urine in animals subjected to the same treatment and withdrawal period might be related to individual characteristics. Although certain variations in the concentrations can be observed, such differences in oxytetracycline pharmacokinetics have been previously described [47].

The half-life time calculated using Equation (1) for oxytetracycline in urine was 4.18 days (Equation (1), Table 1). Considering the compound’s high initial concentration, and extrapolating from the regression calculating the time required for the entire excretion of the antimicrobial from urine, more than two months would be needed to attain a complete excretion, a much longer period when compared to the end of the withdrawal period set by the manufacturer (28 days). This could be one of the reasons why this antimicrobial family is frequently detected at high levels in farm wastes [48], along with the fact that waste treatments are often either poor or non-existent. Tetracyclines have indeed been detected in groundwaters and lagoons in the vicinity of swine production facilities [49,50,51].

### 2.3. Excretion of Enrofloxacin via Urine

Enrofloxacin absorption is nearly complete after intramuscular administration, but to a great extent it is metabolized to ciprofloxacin. This is the reason why both compounds need to be explored via analysis. Enrofloxacin is mainly excreted via urine, with concentrations several times higher than in blood plasma; only slight amounts appear in faeces [10].

**Figure 3:**
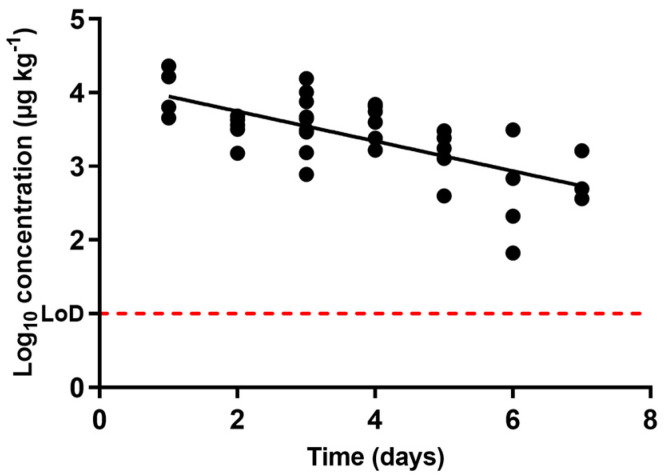
Occurrence of the concentration of enrofloxacin in urine samples obtained from pigs treated with enrofloxacin at pre-set intervals within the withdrawal period (when day 0 matches the end of the treatment), determined via liquid chromatography with tandem mass spectrometry (LC-MS/MS). The LoD dotted line represents the detection limit of the analytical technique for enrofloxacin.

Enrofloxacin concentrations described in urine through the withdrawal period are presented in Figure 3. As with the other two antibacterial compounds, enrofloxacin concentrations declined as the withdrawal period progressed, and concentration decrease was an exponential function of time. From the regression over the data obtained, the compound’s half-life time was found to be 1.48 days (Table 1).

Apart from half-life times, Table 1 shows the regression’s goodness of fit. Once again, a low *R^2^* value was found, as differences could be observed between the concentrations described in different animals subjected to the same treatment and withdrawal period. Nonetheless, such divergences have been previously demonstrated for other antimicrobials and drugs in general. In particular, differences in enrofloxacin metabolization have been previously described in plasma after oral [52] and subcutaneous [53] administration in pigs.

The regression also allowed us to estimate the time period after the end of treatment for the complete excretion of enrofloxacin: almost three weeks. As occurred with oxytetracycline, pigs would actually continue excreting enrofloxacin long after the end of the withdrawal period set by the manufacturer (12 days). This means that although antimicrobials might have been removed from muscle, they are still being disseminated by previously treated animals and should be taken into consideration in terms of farm waste management.

### 2.4. Comparative Study

**Figure 4:**
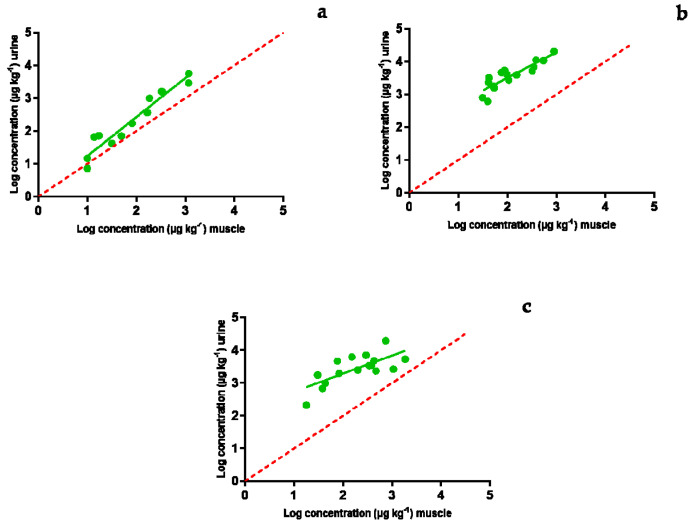
Relationship among the concentrations of sulfamethoxypyridazine (**a**), oxytetracycline (**b**), and enrofloxacin (**c**) detected in muscle and urine samples. The bisecting dotted line represents the 1:1 correlation if both matrixes contained the same concentration of enrofloxacin.

Urine samples were obtained in parallel with muscle and blood samples gathered for the study performed by Serrano et al. [54]. That study’s aim was to find a suitable matrix for *ante mortem* tests for the detection of antimicrobial compounds, which would avoid the slaughter of contaminated animals and thus the entrance of antimicrobial compounds into the food chain. The matrix should be characterized via an easy extraction procedure from living animals and a good correlation with the concentration found in muscle. Whilst blood proved to fulfil both requirements [54], urine results obtained in the present study showed that urine is not a suitable matrix for that purpose (Figure 4). These findings agree with those described previously by Seymour et al. [55] for cows: urine samples from treated cows tested positive for the presence of antimicrobial residues even after the end of the withdrawal period set by the manufacturer. Moreover, this issue could also be interpreted as the occurrence of higher levels of antimicrobials in urine compared to other tissues such as muscle.

Figure 4 describes the correlation between the concentration of sulfamethoxypyrydazine (Figure 4a), oxytetracycline (Figure 4b), and enrofloxacin (Figure 4c) in muscle and urine. With the exception of sulfamethoxypyrydazine, concentrations described in urine were much higher than the ones described for muscle. This indicates that antibiotics might be eliminated for periods of time longer than those required by the European maximum residue limits for muscle [56]. Higher concentrations in urine compared to muscle are common and have been previously described for several antimicrobials such as oxytetracycline, florfenicol, tylosin, sulfadiazine, trimetropim, and enrofloxacin in food-producing swine [13].

Although the high concentrations described in urine open up new possibilities for urine applications in the field of detection tests for antimicrobial residues (for instance, to detect illegal treatments or to ensure that a production system remains free of antibiotics), they also point to the imperative need of controlling farm residues, as most of the antimicrobials removed by urine remain unaltered or are transformed into metabolites of the administered compound [49,50]. Antimicrobials used in this study are between the most commonly used in Europe and are included in novel studies about soil vulnerability to antibiotic contamination [57]. Fertilization of fields with manure and slurry could spread these residues to the fields close to farms when fertilizing them. This practice is increasingly considered a concern [58], mainly due to the impact on natural microbial communities [58,59,60] and the potential spread of antibiotic resistant bacteria and antibiotic resistance genes [58,61]. Moreover, antimicrobial residues reach surface waters and groundwater by means of leaching [62], reaching distant areas and even water used by industry or for human supply. In an attempt to tackle this problem, the EMA has developed a guideline on environmental impact assessment of new veterinary pharmaceuticals [63]. This guide proposes that the estimation of the total release of antimicrobials is used as the key to assess their safety, following a similar philosophy as the one performed in the current study. Nonetheless, good management of slurry is essential in order to deal with the problem of uncontrolled antimicrobial delivery, but as not all the antimicrobials present the same elimination rates, a good knowledge on the subject of their excretion is thus required to improve waste treatment.

**Figure 5:**
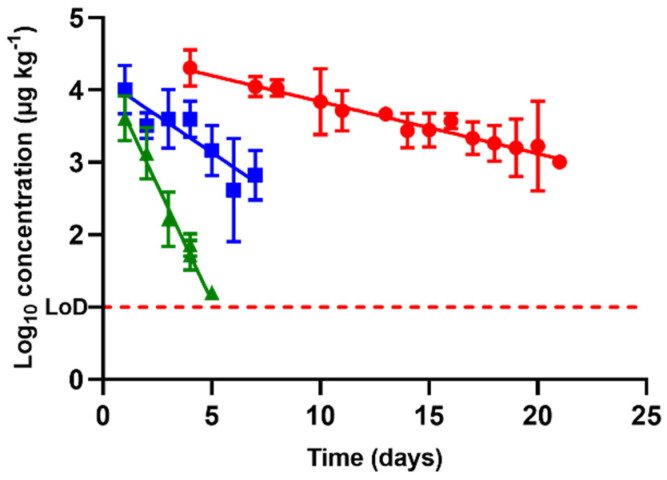
Comparison between the concentrations and rates of removal found for sulfamethoxypyridazine (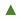), oxytetracycline (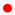), and enrofloxacin (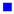) in urine after animal treatment within the withdrawal period. The LoD dotted line represents the detection limit of the analytical technique for sulfamethoxypyridazine, oxytetracycline, and enrofloxacin.

Referring back to antimicrobial excretion, the concentration in urine followed an exponential rate of elimination regardless of the antimicrobial tested (Figure 5). Nevertheless, among compounds, depletion rates differed (*p* < 0.05), and thus the concentrations measured each day differed as well. The highest concentrations at the beginning of the withdrawal period were described for oxytetracycline (data obtained from the regression). This circumstance, along with the slowest rate of removal described in this study (half-life of 4.18 days, Table 1), indicates that this compound might be a problematic antibacterial residue in terms of farm waste management.

Enrofloxacin had a half-life of 1.48 days and sulfamethoxypyridazine of 0.49 days (Table 1). Thus, considering the extended half-life of oxytetracycline, urine removal rates described by these antimicrobials differed by up to nearly 10 times. Good knowledge regarding their excretion is thus critical in order to avoid uncontrolled delivery of antibacterial compounds to a farm’s surroundings.

As defined by the European Medicine Agency [6], sulfonamides and tetracyclynes belong to category D in the classification of antibiotic classes for veterinary use, which means that they should be the first treatment choice. Meanwhile, enrofloxacin pertains to category B; it is of major relevance in human medicine and should be used only when no equivalently effective antimicrobials in categories C or D can be found. These considerations are nevertheless restricted to the EMA’s actual recommendations regarding a responsible use of antimicrobials, designed to encourage veterinarians to carry out an antibiogram before prescribing an antimicrobial treatment.

Thus, in the case of equal sensitivity to the antimicrobials studied, sulfamethoxypyrydazine and oxytetracycline would be the treatments to suggest. In terms of waste management and in view of the data presented in this study, sulfamethoxypyridazine would be the ideal one of the two, as it is more rapidly eliminated in urine. This fast excretion rate implies short periods of extra waste treatment, leading to energetic reduction and economic savings while minimizing the delivery of antibacterial residues to the environment. However, the increase in antimicrobial resistances investigated in recent years [64] and the widespread description of sulfonamide resistance in different niches such as wastewaters, sewage, aquaculture, compost, and even the meat food supply chain [65,66,67] might make these compounds useless against an outbreak etiological agent [68,69], thus making it necessary to give preference to another antibiotic instead. In case such a situation arises, and for purposes of responsible waste management, the sequence of selection would be proportional to the rate of removal: antimicrobials with faster rates of removal would be the therapeutics of choice.

## 3. Materials and Methods

### 3.1. Urine Samples

#### 3.1.1. Antimicrobials

The antimicrobials selected for animal treatment were sulfamethoxypyridazine (a sulfonamide), oxytetracycline (a tetracycline), and enrofloxacin (a quinolone) as they are among the most widely used antimicrobials in EU farming production; they follow different metabolic pathways [70], and they are all effective against a wide range of bacteria. Each of these compounds was administered via intramuscular (IM) injection. Table 2 shows the main features of the antimicrobial compounds used.

#### 3.1.2. Collection of Urine Samples Containing In Vivo Injected Antibiotics

A total of 66 previously untreated piglets were procured at treatment onset. Starting at 40 days before administration of the compounds, they were kept on the premises of the Faculty of Veterinary Sciences at the University of Zaragoza (Zaragoza, Spain) and used in tandem for another study [54]. Pigs were provided by a commercial farm (Valporgen S.L., Zaragoza, Spain), their genetics were Landrace × Large White, and they weighed an average of 44.9 ± 4.8 kg at reception. From them, 39 were female and 27, male. During acclimatization and withdrawal periods, the piglets were fed *ad libitum* with a special mixed feed free of antibiotics (ARS Alendi S.A., Huesca, Spain), and water was provided from a separate, controlled water circuit. Piglets were raised in separate pens depending on the antibiotic with which they were administrated. Table 2 summarizes the main characteristics of the treatments carried out with each antimicrobial.

Overall, 4 pigs remained untreated as blank sample references, and 62 pigs were treated: 20 were administered with sulfamethoxypyridazine, 20 with oxytetracycline, and 22 with enrofloxacin. Urine was collected on different days within the withdrawal period determined by the respective antimicrobial drug manufacturers. It was collected from live pigs via mobilization of the animal and bladder stimulation. At pre-set time intervals, pigs were slaughtered and urine was obtained via bladder puncture; at the same time, urine was gathered from living animals subjected to the same treatment and withdrawal period. Consequently, urine was collected from 1 to 9 pigs on each day of analysis. As animals were bred in batches and slaughtered all along the withdrawal period, the number of sampled animals decreased as the withdrawal period progressed. Samples were kept aliquoted and frozen at −20 °C until analysis.

#### 3.1.3. Ethical Approval

The study was carried out in accordance with the ARRIVE (Animal Research: Reporting of In Vivo Experiments) initiative and approved by the Animal Ethics Experimentation Committee of the University of Zaragoza (PI58/17). Animals were handled and used in accordance with the Spanish Animal Protection Policy RD 53/2013 [71], which complies with the European Union Directive 2010/63 [72] on the protection of animals used for experimental and other scientific purposes.

### 3.2. LC-MS/MS Analysis

#### 3.2.1. Standards and Reagents

LC-MS/MS grade solvents were from Fisher Chemical (Fisher Scientific, Leics, UK). Formic acid (98–100%) was from Fisher Chemical (Fisher Scientific, Geel, Belgium). Purified water was obtained through a Milli-Q system (Millipore, Merck KGaA, Darmstadt, Germany). Ciprofloxacin, enrofloxacin, oxytetracycline, and sulfamethoxypyridazine, as well as internal standard (IS) demeclocycline, were made by Vetranal (Sigma-Aldrich AG, Buchs, Switzerland). Enrofloxacin-d8 and sulfamethoxypyridazine-d3 were purchased from Witega (Witega, Berlin, Germany). For the preparation of 0.1 M EDTA, 3.72 g of EDTA Na_2_·H_2_O (>98%, Sigma-Aldrich Chemie, Steinheim, Germany) were dissolved and mixed with up to 100 mL of distilled water.

Stock solutions (1 mg mL^−1^) for each standard were prepared in methanol and kept at −20 °C. Spiking solution contained each of the studied analytes at 0.6 µg mL^−1^, and IS spiking solution contained 1.2 µg mL^−1^ of each IS. Both solutions were prepared in methanol and maintained at −20 °C.

#### 3.2.2. Sample Preparation

1.5 mL of urine were spiked with 100 µL of IS spiking solution. 100 µL 0.1M EDTA and 5 mL of purified water were added and mixed. 500 µL were taken and further diluted with 1500 µL of purified water. It was filtered through 0.2 µm directly into HPLC vials.

#### 3.2.3. LC-MS/MS Determination

All urine samples were analysed using a SCIEX Exion LC coupled to a TripleQuad 6500+ triple quadrupole detector equipped with an Acquity UPLC^®^ BEH C18 column (1.7 µm, 2.1 × 100 mm). The mobile phase consisted of eluent A (0.1% formic acid in water) and eluent B (0.1% formic acid in acetonitrile) at a flow rate of 0.4 mL min^−1^. The gradient started at 5%B, increased at a constant rate until 40%B in 3.75 min, then increased in a second ramp until 95%B in 4.37 min, held constant until 5.00 min, then returned to the initial 5% and held constant until 7.00 min. The injected volume was 5 µL. Positive electrospray ionisation was used, and analytes were detected in MRM mode by monitoring 2 transitions for each compound of interest (Table 3). Regardless of the compound analysed, the technique’s limit of detection (LoD) was 10 ppb.

### 3.3. Pharmacokinetic Parameters and Statistical Analysis

For each day of the withdrawal period pre-set in this study to evaluate the behaviour of antimicrobials, 1 to 9 samples of urine coming from different animals subjected to the same treatment and withdrawal period were analysed via LC-MS/MS. Results were represented as the mean ± standard deviation using the PRISM^®^ program (GraphPad Software, Inc., San Diego, CA, USA).

The rates of elimination (*λ_z_*) of antibiotics from urine were determined via regression analysis. The corresponding half-lives of elimination(*T_1/2_*) were calculated according to the following equation:

Equation (1): Half-life of elimination where $T_{1/2}$ is the half-life of elimination and $\lambda_{Z}$ is the rate of elimination.

(1) T1/2=Ln (2)λz

Data obtained were analysed and submitted to comparison of averages via ANOVA, followed by a post hoc Tukey test and t-tests with GraphPad PRISM^®^. Differences were considered significant if *p* < 0.05.

## 4. Conclusions

Most of the antibiotics commonly used in chemotherapy are removed from animals´ bodies via urine, thereby contaminating farm wastes, which, if not properly treated, might reach the surroundings, contributing to the dissemination of AMR. To avoid this, a good choice of antimicrobial compound for therapy is required, along with accurate waste management. The study of three widely used antimicrobials showed high excretion rates via urine. This finding has great potential in terms of antibiotic control on the farm level, although it necessarily requires that producers perform slurry purification. Sulfamethoxypyridazine proved to be rapidly excreted by urine (a half-life time of 0.49 days) and enrofloxacin had an intermediate rate of removal by urine (1.48 days); conversely, oxytetracycline took much longer (4.18 days). Hence, after the study of these compounds’ removal rates, at equal susceptibility of the target microorganism, sulfamethoxypyridazine would be the treatment of choice, proving that an in-depth study of the excretion pathways of antimicrobials used in farms will help farmers and producers to choose the most appropriate one in order to optimize waste management and avoid the emergence of AMR while preserving the global One Health approach.

## Figures and Tables

**Table 1:** Half-lives of elimination (*T*_1/2_) calculated using Equation (1) and estimated times for the complete removal from urine determined for sulfamethoxypyridazine, oxytetracycline, and enrofloxacin expressed in days.

Antibacterial Molecule	Slope	^1^ *R^2^*	^2^ *λ_z_*	*T*_1/2_	Estimated Time for Complete Excretion
Sulfamethoxypyridazine	0.6144 ± 0.1052	0.99	0.71	0.49	6.89
Oxytetracycline	0.07194 ± 0.015	0.64	6.04	4.18	63.38
Enrofloxacin	0.2027 ± 0.0760	0.47	2.14	1.48	20.48

^1^*R^2^* coefficient describes the goodness of the fit found for the occurrence of the antimicrobial´s removal by urine. ^2^ *λ_Z_*: rate of elimination.

**Table 2:** Source and main characteristics of the antimicrobials explored. Both administration patterns and withdrawal periods are recommended by the manufacturer of the veterinary products.

Group	Active Compound	Commercial Name	Trading Company	Administration Pattern	Withdrawal Period (Days)
Sulfonamide	Sulfamethoxypyridazine	SULFAMETOX	S. P. VETERINARIA (Tarragona, Spain)	Attack dose of 40 mg kg^−1^ Maintenance dose 20 mg kg^−1^ for 5 days	28
Tetracycline	Oxytetracycline	ALAMYCIN L.A 300	KARIZOO LAB (Barcelona, Spain)	Single dose of 30 mg kg^−1^	28
Quinolone	Enrofloxacin	BAYTRILUNO 100 mg mL^−1^	BAYER (Leverkusen, Germany)	2 doses of 7.5 mg kg^−1^ separated 48 h	12

**Table 3:** Monitored ions via LC-MS/MS ESI+ in MRM mode.

Compound	Precursor	Product	^1^ DP (V)	^2^ CE (V)
Enrofloxacin	360	342	72	30
		266	72	50
Ciprofloxacin	332	314	61	30
		231	61	50
Ciprofloxacin-D8 (IS)	340	322	61	30
Sulfamethoxypyridazine	281	156	60	25
		108	60	35
Sulfamethoxypyridazine-D3 (IS)	284	156	60	25
Oxytetracycline	461	426	65	30
Demeclocycline (IS)		443	65	17
	465	154	65	40

^1^ DP: declustering potential; ^2^ CE: collision energy.

## Data Availability

Data is contained within the article.
